# Identifying Phage Virion Proteins by Using Two-Step Feature Selection Methods

**DOI:** 10.3390/molecules23082000

**Published:** 2018-08-10

**Authors:** Jiu-Xin Tan, Fu-Ying Dao, Hao Lv, Peng-Mian Feng, Hui Ding

**Affiliations:** 1 Key Laboratory for Neuro-Information of Ministry of Education, School of Life Science and Technology, Center for Informational Biology, University of Electronic Science and Technology of China, Chengdu 610054, China; tjx0705@163.com (J.-X.T.); koyee_d@sina.com (F.-Y.D.); 13208188368@163.com (H.L.); 2 Hebei Province Key Laboratory of Occupational Health and Safety for Coal Industry, School of Public Health, North China University of Science and Technology, Tangshan 063000, China

**Keywords:** phage virion protein, feature fusion, ANOVA, mRMR, machine learning

## Abstract

Accurate identification of phage virion protein is not only a key step for understanding the function of the phage virion protein but also helpful for further understanding the lysis mechanism of the bacterial cell. Since traditional experimental methods are time-consuming and costly for identifying phage virion proteins, it is extremely urgent to apply machine learning methods to accurately and efficiently identify phage virion proteins. In this work, a support vector machine (SVM) based method was proposed by mixing multiple sets of optimal g-gap dipeptide compositions. The analysis of variance (ANOVA) and the minimal-redundancy-maximal-relevance (mRMR) with an increment feature selection (IFS) were applied to single out the optimal feature set. In the five-fold cross-validation test, the proposed method achieved an overall accuracy of 87.95%. We believe that the proposed method will become an efficient and powerful method for scientists concerning phage virion proteins.

## 1. Introduction

The bacteriophage, which is also known as the phage, is a kind of virus that infects bacteria and can complete growth and reproduction in bacteria [1]. The bacteriophage consists of an outer protein coat and a single genetic material (DNA or RNA) inside [2] and is the most widely distributed group of the virus, which is usually found in places full of bacterial communities such as soil and animals’ intestine.

Proteins are the major components of viruses including structural proteins (namely, phage virion proteins) and non-structural proteins (namely, phage non-virion proteins). Non-structural proteins are proteins encoded by viral genes that function in the process of viral gene expression, but they don’t bind to viral particles. Structural proteins are the necessary proteins to form mature and infectious virus particles including shell proteins, envelope proteins, and virus particle enzymes, etc. Among them, the shell proteins are the proteins that constitute the structure of the virus capsid and their main function is protecting the viral nucleic acid, participating in the adsorption and invasion of bacteriophages, and more. Envelope proteins are the proteins that constitute the viral envelope structure and the main function is to act as a viral surface antigen, which maintains the viral structure, participates in virus budding, and more. Due to the clear differences in the function of structural proteins and non-structural proteins, the correct identification of them will be helpful to further understand the molecular mechanisms of bacteriophage genetics and the development of antimicrobial drugs.

The traditional method for the identification of the phage virion and non-virion proteins is mass spectrometry (MS) [3]. However, it has not kept pace with the explosive growth of protein sequences in the post-genome era. Therefore, it is necessary to adopt machine learning methods to identify phage virion proteins. In 2013, Feng et al. proposed a Naïve Bayes classifier to identify phage virion proteins [4]. Afterward, Ding et al. developed an SVM-based method to identify phage virion proteins in which the proteins were encoded using the optimal features obtained by using the ANOVA feature selection technique [5]. In 2015, Zhang et al. introduced an ensemble method for predicting phage virion proteins from phage protein sequences by combining the CTD, bi-profile Bayes, PseAAC, and PSSM [6]. Subsequently, Manavalan et al. proposed a method called PVP-SVM, which adopted the SVM classifier with multiple feature extraction methods [7]. More recently, Fan et al. proposed a novel method called PhagePred to predict phage virion proteins by using the Multinomial Naïve Bayes classifier combined with the g-gap features tree [8]. However, these current feature extraction methods do not describe the protein sequences completely. Therefore, it is necessary to apply new feature extraction and selection methods to investigate the identification of phage virion proteins further.

**Figure 1:**
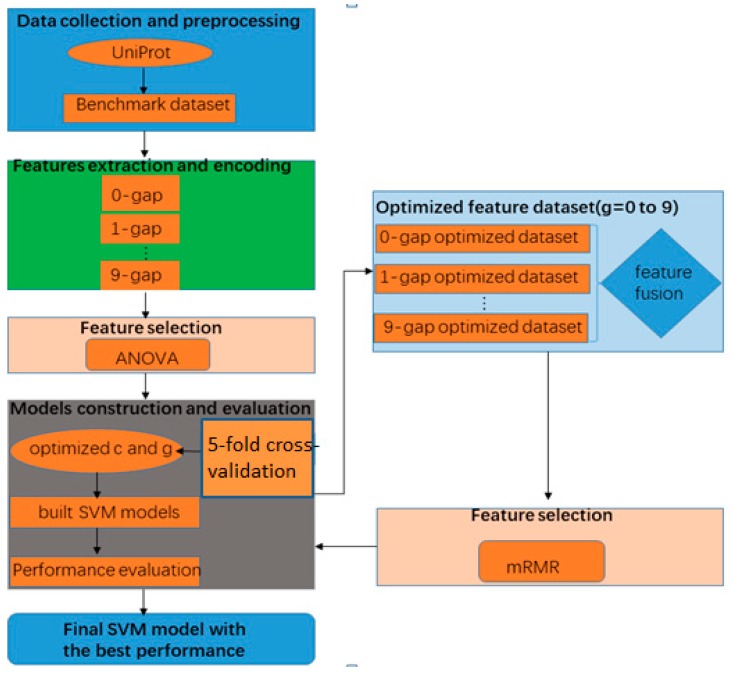
The framework of the proposed method.

In this paper, by using a new feature extraction method and two feature selection methods (ANOVA and mRMR) to select optimal features, we proposed an SVM-based method to identify phage virion proteins. As shown in Figure 1, the rest of the paper is organized based on the following aspects: (1) collection of raw data sets and processing of raw data sets, (2) feature extraction, (3) feature selection, (4) classifier algorithm, and (5) performance evaluation.

## 2. Materials and Methods

### 2.1. Benchmark Dataset

The raw positive and negative datasets adopted in this research were extracted from the Universal Protein Resource (UniProt) [9]. In order to obtain a high-quality and reliable benchmark dataset, the following steps were performed. First, only the phage virion proteins and phage non-virion proteins that have been experimentally confirmed can be included. Second, the protein sequences that are fragments of other proteins were excluded. Third, protein sequences containing nonstandard letters such as ‘B,’ ‘U,’ ‘X,’ or ‘Z’ were eliminated because their meanings are ambiguous. By following these three rigorous screening processes, a total of 121 phage virion protein sequences and 231 phage non-virion protein sequences were obtained. In order to obtain a high quality benchmark dataset, the CD-HIT [10] program was used by setting the cutoff threshold of the protein sequence identity to 40%. Lastly, we obtained 99 phage virion protein sequences and 208 phage non-virion protein sequences.

It is necessary to evaluate the proposed model by using an independent dataset to check whether the prediction model has a generalization capability. In this study, the independent dataset constructed by Manavalan et al. [7] was used, which can be downloaded from http://www.thegleelab.org/PVP-SVM/SVM-PVPData.html. It is a reliable independent dataset containing 30 phage virion protein sequences and 64 phage non-virion protein sequences.

### 2.2. The g-Gap Dipeptide Composition

After the benchmark dataset was built, we needed to describe the protein sequences using a computer-readable form. Proteins are formed with the use of 20 amino acids, according to a certain order and space structure. The most common and simplest method is to formulate the sample protein **P** with *L* residues with its entire amino acid sequence, which is shown below.

(1) $$P=A_{1}A_{2}A_{3}\ldots A_{L}$$

where $A_{1}$ represents the first amino acid residue of the sample protein **P**, $A_{2}$ represents the second amino acid residue of the sample protein **P**, and so forth.

Another straightforward method to formulate the protein sequence is amino acid composition (AAC). In order to obtain the sequence-related information, the AAC is replaced by the adjacent dipeptide composition to represent the protein sequences [4]. However, the adjacent dipeptide composition can only express the correlation between two adjacent amino acid residues. In fact, in three-dimensional space, two amino acids with *g*-gap residues may be adjacent. In order to find the important correlation in protein sequences, we applied the *g*-gap dipeptide composition, which is extended from the adjacent dipeptides [8,11,12] and is a kind of mode of PseAAC [13,14,15]. By adopting this method, a sample protein **P** can be formulated by using the equation below.

(2) $$P={\left[v_{1}^{g},v_{2}^{g},\ldots ,v_{i}^{g},\ldots v_{400}^{g}\right]}^{T}$$

where the symbol *T* represents the transposition of the vector while the $v_{i}^{g}$ represents the frequency of the *i*-th (*i* = 1, 2, …, 400) *g*-gap dipeptide and can be formulated by using the equation below.

(3) $$v_{i}^{g}=\frac{n_{i}^{g}}{L-g-1}$$

where $n_{i}^{g}$ represents the number of the *i*-th *g*-gap dipeptide, *L* represents the length of the protein **P**, *g* represents the number of amino acid residues separated by two amino acid residues, *g* = 1 indicates the correlation between two amino acid residues with the interval of one residue, *g* = 2 indicates the correlation between two amino acid residues with the interval of two residues, and so forth.

### 2.3. The Analysis of Variance (ANOVA)

In general, if a model was built on a low-dimensional feature subset, the robustness of the model will be excellent. However, the low dimensionality of the feature subset will not provide enough information, which results in a poor performance of the model. On the contrary, a high-dimensional features subset can lead to information redundancy and overfitting problems. Both of these problems will lead to low accuracy of the cross-validation prediction and a poor predictor generalization ability. In order to overcome these shortcomings, the best way is to pick out the best feature subset, but it is time-consuming to investigate the performance of all feature subsets by the computer [16,17,18,19]. For example, if the amino acid composition contains a 400-dimension feature vector, the number of all possible combinations for the 400-D vector is $C_{400}^{1}+C_{400}^{2}+C_{400}^{3}+\ldots +C_{400}^{399}+C_{400}^{400}=2.58\times 10^{120}$. Therefore, the analysis of variance (ANOVA) method with the incremental feature selection (IFS) [20,21,22] process was applied to investigate the optimal feature set with the maximum accuracy.

The ANOVA method can score each feature according to a unified standard and then reduce the features according to their contribution. This will not only save the calculation time but will also improve the model’s performance. According to the principle of ANOVA, the score (F) of the *i*-th *g*-gap dipeptide can be formulated by using the formula below.

(4) $$F\left(i\right)=\frac{S_{B}^{2}\left(i\right)}{S_{W}^{2}\left(i\right)}$$

where $S_{B}^{2}\left(i\right)$ represents the variance between groups (MSB) of *i*-th feature in the sample and $S_{W}^{2}\left(i\right)$ represents the variance within groups (MSW) of *i*-th feature in the sample, which are calculated by using the equations below.

(5) $$\{\begin{array}{c} S_{B}^{2}\left(i\right)=\frac{SS_{B}\left(i\right)}{df_{B}}\\ S_{W}^{2}\left(i\right)=\frac{SS_{W}\left(i\right)}{df_{W}} \end{array}$$

where $df_{B}=K-1$ and $df_{W}=M-K$ represent the degree of freedom for MSB and MSW, respectively. K and M represent the number of group (here *K* = 2) and the number of samples (here *M* = 307), respectively. $SS_{B}\left(i\right)$ and $SS_{W}\left(i\right)$ represent the sum of MSB and MSW, respectively, and are calculated by using the formula below.

(6) $$\{\begin{array}{c} SS_{B}\left(i\right)=\overset{2}{\underset{j=1}{\sum }}m_{j}{\left(\frac{\sum_{s=1}^{m_{j}}f_{i}^{g}\left(s,j\right)}{m_{j}}-\frac{\sum_{j=1}^{2}\sum_{s=1}^{m_{j}}f_{i}^{g}\left(s,j\right)}{\sum_{j=1}^{2}m_{j}}\right)}^{2}\\ SS_{W}\left(i\right)=\overset{2}{\underset{j=1}{\sum }}\overset{m_{j}}{\underset{s=1}{\sum }}{\left(f_{i}^{g}\left(s,j\right)-\frac{\sum_{s=1}^{m_{j}}f_{i}^{g}\left(s,j\right)}{m_{j}}\right)}^{2} \end{array}$$

where $f_{i}^{g}\left(s,j\right)$ represents the frequency of the *i*-th *g*-gap dipeptide of the *j*-th sample in the *s*-th group. 

A feature with a high F(i)-value means that its ability to identify the sample is excellent, which is more conducive to building a highly robust model. Therefore, we ranked all features according to their F(i)-values from high to low and obtained new feature vectors, which are shown below.

(7) $$P_{g}^{\prime }={\left[v_{1,g}^{\prime },v_{2,g}^{\prime },\ldots ,v_{i,g}^{\prime },\ldots v_{n,g}^{\prime }\right]}^{T}\left(0\leq g\leq 9\right)$$

By using the ANOVA method, we have a clear understanding of each feature’s capabilities for the model. Therefore, we don’t have to exhaust all the feature subsets but instead selectively construct feature subsets according to their F(i)-values in this paper. The first feature subset contains the feature with the highest F(i)-value, $P_{g}^{\prime }=\left[v_{1,g}^{\prime }\right]$. The second feature subset adds the second highest F(i)-value to the first subset, $P_{g}^{\prime }=\left[v_{1,g}^{\prime },v_{2,g}^{\prime }\right]$. The third feature subset adds the third highest F(i)-value to the second subset, $P_{g}^{\prime }=\left[v_{1,g}^{\prime },v_{2,g}^{\prime },v_{3,g}^{\prime }\right]$. The procedure was repeated until the accuracy of the model no longer increased.

### 2.4. Minimal-Redundancy-Maximal-Relevance (mRMR)

The combination of some of the best-performing features does not mean that the best predictive effect can be achieved. The main reason for this phenomenon is that these features are likely to have a high degree of correlation, which leads to more redundant information in the feature vector. To solve this problem, Peng et al. proposed the mRMR algorithm [23]. MRMD [24] is another tool similar to mRMR. The main idea of the algorithm is to filter out some of the most relevant features in the subset to achieve the goal of minimizing information redundancy and then obtain the most ‘concise’ subset of features in theory. Therefore, when using the mRMR-ranked feature benchmark dataset with a smaller dimension, it can still effectively represent a dataset with a larger dimension, which can ensure that the feature dimension and the time of the training model are greatly reduced with almost no loss of effective information.

The mRMR algorithm is often used to select discretization features and continuity features. Based on the issues involved in this paper, the following is a description about discretization features. Given two random discrete variables *x* and *y*, the mutual information I(x,y) between them can be calculated by using the formula below.

(8) $$I\left(x,y\right)=\iint p\left(x,y\right)log\frac{p\left(x,y\right)}{p\left(x\right)p\left(y\right)}dxdy$$

where the mutual information I(x,y) is a measure of the degree of correlation between two random variables *x* and *y* and p(x),p(y),p(x,y) denote the probabilistic density functions, respectively. The metrics of the mRMR algorithm are Max-Relevance and Min-Redundancy and they can be described by the equations below.

1. Max-Relevance:
  (9) $$max\;D\left(S,c\right),D=\frac{1}{\left|S\right|}\underset{x_{i}\in S}{\sum }I\left(x_{i};c\right)$$
2. Min-Redundancy:
  (10) $$min\;R\left(S\right),R=\frac{1}{{\left|S\right|}^{2}}\underset{x_{i},x_{j}\in S}{\sum }I\left(x_{i};x_{j}\right)$$
  where $x_{i}$ represents the *i*-th feature attribute, *c* represents the category variable, *S* represents the feature subset, and |S| represents the size of the feature subset. Furthermore, if the two metrics are considered equally important, we can abbreviate it by using the formula below.
  (11) $$\{\begin{array}{c} max\left(D_{I}-R_{I}\right)\\ min\left(\frac{D_{I}}{R_{I}}\right) \end{array}$$

### 2.5. Support Vector Machine (SVM)

The support vector machine (SVM) is a widely used binary classification model, which has been widely used in bioinformatics [25,26,27,28,29,30,31,32,33,34,35,36,37,38]. It is a supervised machine learning method and its main idea is to map the input features from low-dimensional space to a high-dimensional space through nonlinear transformation and then find the optimal linear classification surface in this high-dimensional space. For convenience, SVM software packages LibSVM can be download from https://www.csie.ntu.edu.tw/~cjlin/libsvm/. In the current study, the LibSVM package was adopted to investigate the performance of identifying the phage virion proteins. Since it has been widely adopted in bioinformatics, the radical basis function kernel was selected to perform predictions. 

### 2.6. Performance Evaluation

Three cross-validation methods, which are called the independent dataset test, the sub-sampling test, and the jackknife test, are widely used to evaluate the predictive ability of a predictor in practical application [4,28,39,40,41,42,43,44,45,46,47,48,49]. Among these three methods, the jackknife test was considered to be the most rigorous one that can get a unique outcome in statistical prediction. This test has been widely used by investigators to assess the performance of the predictor [4,37,46,50,51,52,53,54]. In this paper, in order to save computational time, the five-fold cross-validation method was used to tune the parameters *C* and *g* in the SVM.

In this paper, we adopted five evaluation indexes to evaluate the model. Sensitivity ($S_{n}$) is used to evaluate the model’s ability to predict positive samples. Specificity ($S_{p}$) is used to evaluate the model’s ability to predict negative samples. Overall Accuracy (Acc) reflects the proportion of the entire benchmark dataset that can be correctly predicted. The Matthew correlation coefficient (Mcc) is an indicator used to reflect the reliability of the algorithm. Its value is between −1 and 1 and the high value of Mcc indicates that the model has a good prediction performance. The four indexes are defined below.

(12) $$\{\begin{array}{c} \begin{array}{c} S_{n}=\frac{TP}{TP+FN}\\ S_{p}=\frac{TN}{TN+FP} \end{array}\\ \begin{array}{c} Acc=\frac{TP+TN}{TP+FN+TN+FP}\\ Mcc=\frac{TP\times TN-FP\times FN}{\sqrt{\left(TP+FP\right)\left(TP+FN\right)\left(TN+FP\right)\left(TN+FN\right)}} \end{array} \end{array}.$$

where TP, TN, FP, and FN represent the number of the correctly recognized phage virion proteins, the number of the correctly recognized phage non-virion proteins, the number of phage non-virion proteins recognized as phage virion proteins, and the number of phage virion proteins recognized as phage non-virion proteins, respectively.

The ROC (receiver operating characteristic) curve is a more intuitive way to demonstrate the performance of the model. Therefore, we plotted the ROC and calculated the area under the ROC curve (auROC). The high value of auROC indicates that the model has a good classification ability and deserves our trust.

## 3. Result and Discussion

### 3.1. Prediction of Phage Virion Proteins Based on Single Kind of g-Gap Dipeptides 

**Figure 2:**
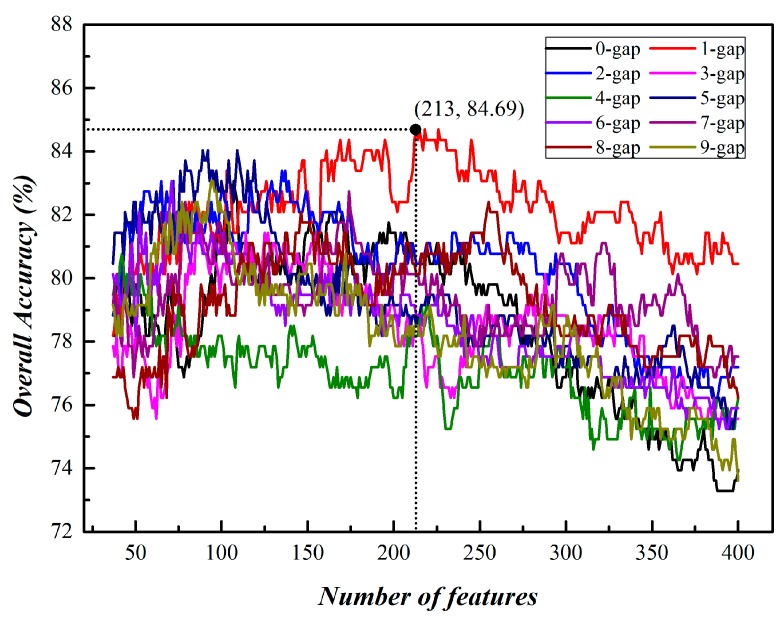
A plot showing the IFS curves for 0-gap to 9-gap.

The ANOVA method with the IFS process was applied to investigate the optimal feature set with the maximum accuracy. The details of the optimization process can be referred to Section 2.3. We chose residue parameter *g* from 0 to 9 to estimate the performance for all the 10×400=4000 feature subsets using SVM until all the 4000 Acc*s* (overall accuracies) were calculated. In addition, we plotted 10 curves by setting the overall Acc as an ordinate and the number of features as abscissa shown in Figure 2. Additionally, the highest predictive accuracies for *g*-gap dipeptides are shown in Table 1.

### 3.2. Prediction of Phage Virion Proteins Based on Fusing Features

For different g (from 0 to 9) values, each optimum subset represents the best characterization of proteins at different levels. If the 10 best feature subsets were fused together, each protein in the benchmark dataset can be encoded using a 107 + 213 + 135 + 87 + 42 + 89 + 70 + 174 + 255 + 94 = 1266-dimensional feature vector, which would be more comprehensive in representing protein sequences. We investigated the performance of identifying phage virion proteins based on the 1266 features. An *Acc* of 85.02% was achieved by adopting SVM in the five-fold cross-validation method. It is worthy of further investigation because the prediction performance was still far from satisfactory.

**Figure 3:**
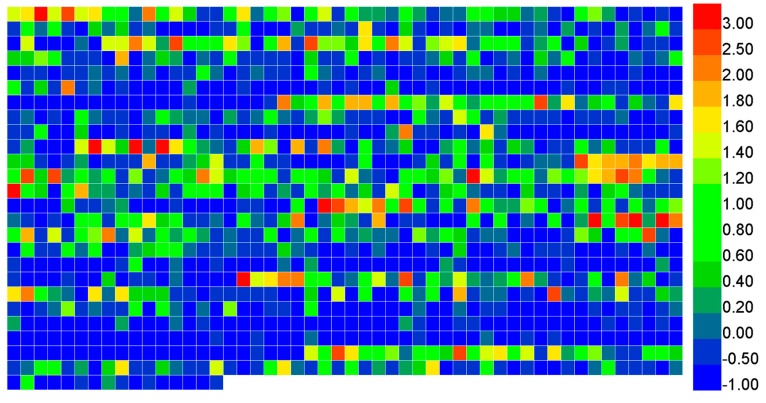
A heat map for 1266 features based on different Z-scores.

However, there exists noise in such a set of features. Therefore, we used the mRMR to score the 1266 features and ranked the features according to their scores. The details of the optimization process is outlined in Section 2.4. In order to show the contributions of each feature to the prediction, we made a heat map for the 1266 features based on their *Z*-scores, which is shown in Figure 3. The scores can be calculated with the help of the formula below.

(13) $$Z_{i}=\frac{x_{i}-\mu }{\sigma }\;(i=1,2,\ldots ,1266)$$

where $x_{i}$ represents mRMR score for the *i*-th features, μ represents the overall average of 1266 mRMR scores, and σ represents the standard deviation of 1266 mRMR scores.

**Figure 4:**
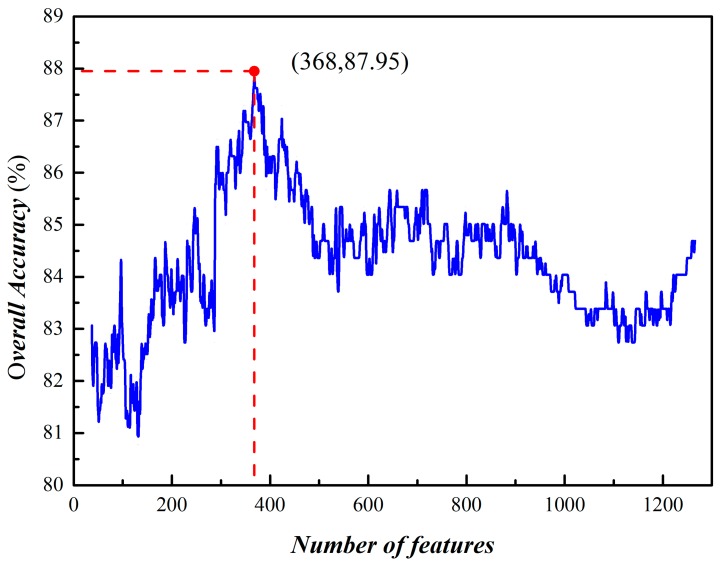
A plot showing the IFS curve by using mRMR.

**Figure 5:**
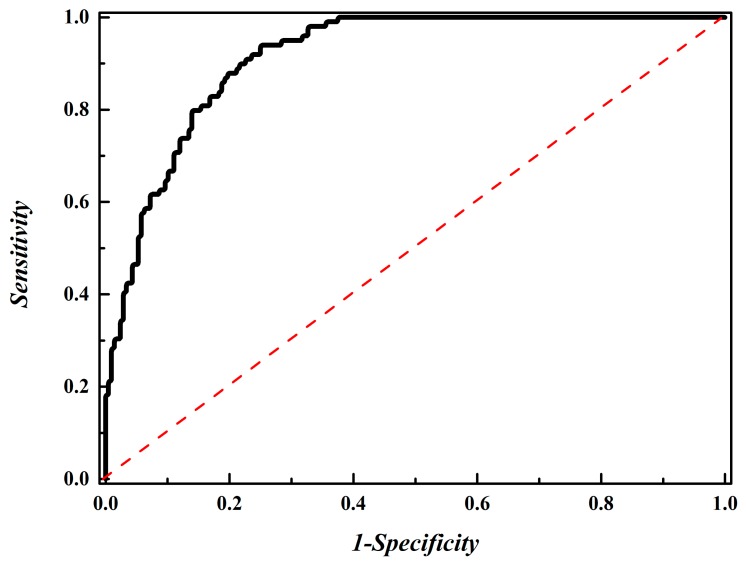
The ROC curve for the prediction of phage virion proteins by using 368 optimal features. The auROC of 0.915 was obtained in a five-fold cross-validation test. The diagonal dot line denotes a random guess with the auROC of 0.5.

By using the IFS process to investigate the performance for all the subsets, the *Acc* reached its peak (87.95%) when the top ranked 368 dipeptides were used to build the model (Figure 4). In this case, the $S_{n}$, $S_{p},$ and Mcc are 83.84%, 89.90%, and 0.761%, respectively, with the auROC at 0.915 (Figure 5). This result indicates that the performance of the proposed model is smart and reliable for identifying phage virion proteins.

### 3.3. Comparision with Other Published Methods

It is necessary to compare the methods used in this article with other published methods. Table 2 shows the detailed predictive results from the published papers. Based on the same benchmark dataset, Feng et al. proposed a Naïve Bayes-based method to predict bacteriophage virion proteins by using the amino acid composition and the dipeptide composition and obtained an overall accuracy of 79.15% [4]. Ding et al. adopted the ANOVA feature selection method to select optimal 1-gap dipeptides and obtained an overall accuracy of 85.02% [5]. Manavalan et al. proposed a novel method called ‘PVP-SVM’ in which the AAC, ATC, CTD, DPC, and PCP were used to represent the protein sequences and got an overall accuracy of 87.0% in the jackknife test [7]. Pan et al. adopted a Multinomial Naïve Bayes classifier based on the discrete features obtained from the *g*-gap feature tree. They achieved a superb overall accuracy of 98.37% in the 10-fold cross-validation [8]. In this work, we achieved an overall accuracy of 87.95%, which is the second-best prediction result until now. Compared to the results with reference [4,5,7] except for the value of $S_{p},$ which is slightly lower than that of Manavalan’s [7], the values of $S_{n}$, Acc, Mcc and auROC are significantly higher than published results. These comparisons demonstrate a better performance of our proposed methods.

### 3.4. Performance Evaluation Using an Independent Dataset

In general, the best model for the training dataset is not the optimal model for the independent dataset. Therefore, we repeated the feature selection procession, retrained the model, and validated the model on an independent dataset. The results for different classifiers were listed in Table 3. As indicated in Table 3, among the three compared methods, our method obtained the highest *S_n_* while the *S_p_*, *Acc*, and *MCC* is similar to the PVP-SVM, which obtained the best predictive results on the independent. This indicates that our method can play complementary roles to existing methods for identifying phage virion proteins.

## 4. Conclusions

In this work, we investigated the accuracies of different features for identifying phage virion proteins. The maximum overall accuracy (87.95%) was obtained by fusing 10 optimal *g*-gap (0 to 9) dipeptide compositions, which was obtained by fusing ANOVA and mRMR feature selection methods. Compared with the existing methods, the proposed model improved the overall accuracy. Therefore, the method can be used as a reliable tool for accurately predicting phage virion proteins. In our study, there are many problems worth investigating for phage virion protein prediction. For example, in order to build a high quality dataset, greater attention should be paid to the dynamic changes of the database. Second, the biological meanings of the selected optimal features also need to be clarified. Third, considering the promising performance of the ensemble classification methods [55] and the deep learning technique [56,57,58] in bioinformatics, we will integrate multiple classification algorithms to build the model for identifying phage virion proteins. User-friendly and publicly accessible web tools including predictors [25,27,50,59,60] or databases [61,62,63,64] represent the future direction for developing a more useful bioinformatics method. In the future, we will establish a powerful tool for phage virion protein prediction. The feature selection strategy can be extended to other fields.

## Figures and Tables

**Table 1:** The maximum *Acc* and the corresponding number of feature at different *g* values.

*g*	Number of Feature	*Acc* (%)
0	107	83.06
1	213	84.69
2	135	83.39
3	87	81.76
4	42	80.78
5	89	84.04
6	70	82.41
7	174	82.73
8	255	82.41
9	94	83.06

**Table 2:** Comparing the proposed method with other published methods.

Ref.	$S_{n}\;(\%)$	$S_{p}\;(\%)$	Acc (%)	Mcc	auROC
[4]	75.76	80.77	79.15	-	0.855
[5]	75.76	89.42	85.02	-	0.899
[7]	73.70	93.30	87.00	0.695	0.900
[8]	96.97	98.56	98.05	0.963	0.990
This work	83.83	89.90	87.95	0.761	0.915

**Table 3:** Comparing the proposed method with other published methods on the independent dataset.

Ref.	$S_{n}\;(\%)$	$S_{p}\;(\%)$	Acc (%)	Mcc	auROC
[5]	60.00	76.50	71.30	0.357	0.742
[7]	66.70	85.90	79.80	0.531	0.844
This work	70.00	78.13	75.53	0.464	0.651
